# Precision pathways: optimising amyloid PET for sustainable Alzheimer’s disease care

**DOI:** 10.1007/s00415-026-13874-2

**Published:** 2026-05-26

**Authors:** Silvia Daniela Morbelli, Marco Bozzali, Annachiara Cagnin, Diego Cecchin, Arturo Chiti, Massimo Filippi

**Affiliations:** 1 Department of Medical Sciences, University of Turin, Turin, Italy; 2 Nuclear Medicine Unit, Azienda Ospedaliero-Universitaria Città della Salute e della Scienza di Torino, Turin, Italy; 3 “Rita Levi-Montalcini” Department of Neuroscience, University of Torino, Turin, Italy; 4 Neurology Unit 2U, Azienda Ospedaliero-Universitaria Città della Salute e della Scienza di Torino, Turin, Italy; 5 Department of Neuroscience, University of Padova, Padua, Italy; 6 Padova Neuroscience Center (PNC), University of Padova, Padua, Italy; 7 Department of Medicine, University of Padova, Padua, Italy; 8 Nuclear Medicine Unit, University-Hospital of Padova, Padua, Italy; 9 Faculty of Medicine and Surgery, Vita-Salute San Raffaele University, Milan, Italy; 10 Nuclear Medicine Department, IRCCS San Raffaele Scientific Institute, Milan, Italy; 11 Neuroimaging Research Unit, Division of Neuroscience, IRCCS San Raffaele Scientific Institute, Milan, Italy; 12 Neurology Unit, IRCCS San Raffaele Scientific Institute, Milan, Italy; 13 Center for Alzheimer’s and Related Diseases (CARD), IRCCS San Raffaele Scientific Institute, Via Olgettina, 60, 20132 Milan, Italy; 14 Neurophysiology Service, IRCCS San Raffaele Scientific Institute, Milan, Italy; 15 Neurorehabilitation Unit, IRCCS San Raffaele Scientific Institute, Milan, Italy; 16 Neurotech Hub, Vita-Salute San Raffaele University, Milan, Italy

**Keywords:** Alzheimer’s disease, Amyloid PET, Anti-amyloid therapy

## Abstract

The approval of anti-amyloid therapies has reshaped Alzheimer’s disease from a clinically defined syndrome to a biologically confirmed and treatment-oriented condition. This transition places biomarkers, particularly amyloid positron emission tomography (Aβ-PET), at the centre of diagnostic precision, patient selection, safety governance, and therapeutic monitoring. Aβ-PET provides high specificity for confirming cerebral amyloid pathology, especially in cases with discordant or inconclusive fluid biomarkers, and supports staging through semi-quantitative assessment using standardised Centiloid metrics. In patients considered for anti-amyloid therapies, baseline Aβ-PET refines eligibility and risk–benefit profiling when integrated with MRI and APOE genotyping. During treatment, longitudinal Aβ-PET enables objective assessment of pharmacodynamic target engagement and treatment-related amyloid clearance, supporting response-adapted strategies and, in selected cases, therapy discontinuation. Beyond its clinical role, Aβ-PET has strategic organisational and economic implications. Its value is maximised when embedded within structured, stepwise diagnostic pathways that use scalable fluid biomarkers for triage and reserve Aβ-PET for high-impact decisions. However, implementation is challenged by regional heterogeneity, capacity constraints, and limited harmonisation across centres. Coordinated hub-and-spoke models, quantitative standardisation, and prospective registry-based data collection are essential to ensure the equitable and sustainable integration of anti-amyloid therapies into clinical practice. Aβ-PET has evolved from a confirmatory diagnostic tool to a strategic instrument that enables biologically driven, outcome-oriented care for patients with Alzheimer’s disease.

## Introduction

β-Amyloid (Aβ) dysfunction and deposition are central pathological hallmarks of Alzheimer’s disease (AD) [1, 2]. For many years, this biological insight of AD was largely confined to research settings, while clinical care focused predominantly on symptomatic management. The approval in Europe in 2025 of anti-amyloid therapies (AATs), which need biological evidence of AD-related neuropathology, represents a substantial shift in the organisation of health services.

Lecanemab and donanemab are monoclonal antibodies targeting various Aβ species. They are the first agents to demonstrate a reproducible association between biological target engagement and clinical benefit in early symptomatic AD. Phase 3 trials [3, 4] have shown that both therapies significantly reduce cerebral amyloid burden and slow cognitive and functional decline in patients with mild cognitive impairment (MCI) or mild dementia due to AD, provided that amyloid pathology is confirmed at baseline. Although the observed clinical effect sizes are modest, corresponding to a relative slowing of disease progression of approximately 25–30%, the change in biological outcomes (i.e., clearance of parenchymal amyloid deposition) is huge. These results mark a paradigm change by establishing disease modification as an achievable therapeutic goal in AD [5].

**Fig. 1:**
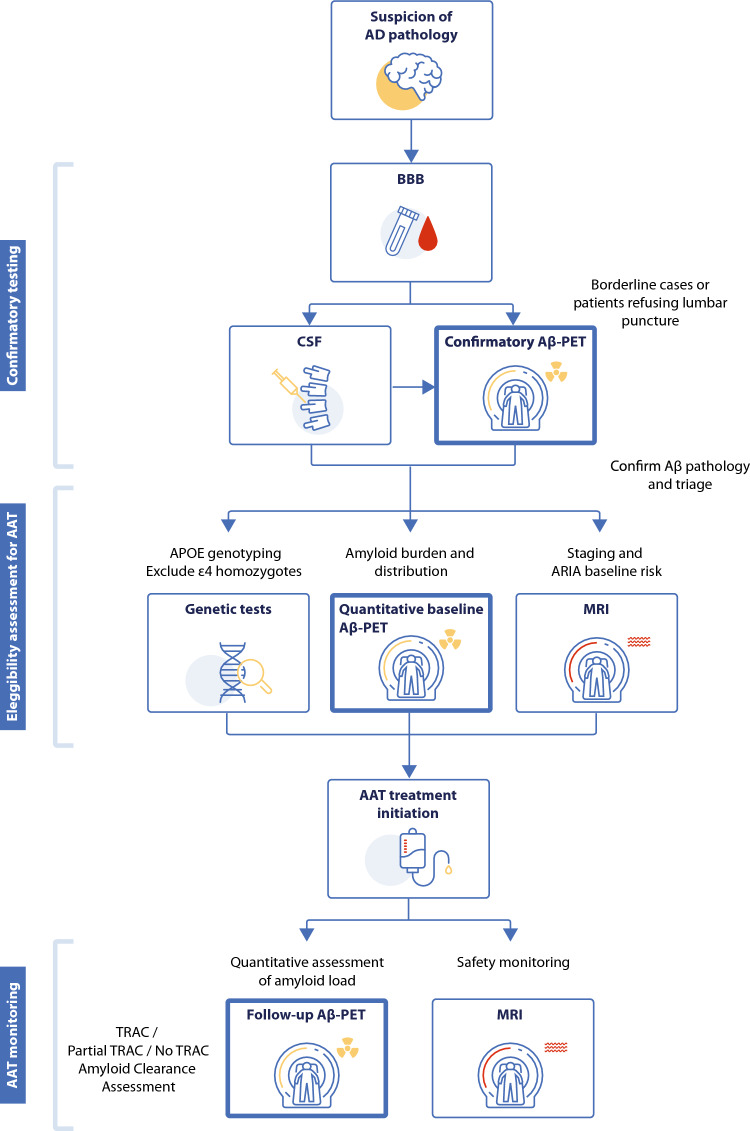
Proposed diagnostic, selection, and monitoring pathway integrating Aβ-PET in the era of anti-amyloid therapy (AAT). Fluid biomarkers (CSF or blood-based) provide first-line confirmation of amyloid pathology, with Aβ-PET reserved for confirmatory assessment in cases that are borderline or inconclusive. MRI supports staging and baseline ARIA risk evaluation, while APOE genotyping refines eligibility. Quantitative Aβ-PET (Centiloids) is performed at baseline, prior to treatment initiation, and at follow-up to assess treatment-related amyloid clearance (TRAC), with MRI used for longitudinal safety monitoring

Despite a shared therapeutic rationale, lecanemab and donanemab differ in target engagement and treatment strategy, with practical implications for clinical workflows. Lecanemab preferentially binds soluble protofibrillar Aβ and is administered as continuous therapy within a longitudinal disease-management framework [4, 6]. In contrast, donanemab targets deposited plaques and incorporates biomarker-guided treatment adaptation, where therapy discontinuation may be considered once predefined amyloid positron emission tomography (Aβ PET) thresholds are met [3]. These differences underscore the central role of biomarkers not only in treatment eligibility but also in therapeutic monitoring and decision-making (Fig. 1).

The introduction of AATs has therefore accelerated the transition from a symptom-based to a biologically defined, outcome-oriented model of care. In this context, biomarkers are essential tools for diagnosis, stratification, and risk–benefit evaluation. Cerebrospinal fluid (CSF) biomarkers provide a well-established measure of amyloid and tau pathology and remain a central component of biological confirmation, particularly when diagnostic certainty is required in specialist settings. Aβ-PET remains a pivotal gold standard for confirming amyloid pathology and supporting high-impact treatment decisions, while emerging blood-based biomarkers (BBB) offer scalable solutions for case-finding and triage within specialist care pathways [7, 8].

At the same time, these AATs introduce new safety considerations, most notably amyloid-related imaging abnormalities (ARIA), which require structured baseline assessment and longitudinal magnetic resonance imaging (MRI) monitoring. While most cases are asymptomatic and resolve on their own, ARIA can manifest as vasogenic oedema (ARIA-E), microhaemorrhages (ARIA-H), or both. In a subset of patients, these changes can be symptomatic, leading to serious neurological events that may require temporary treatment interruption or permanent discontinuation [9]. Optimising the balance between clinical benefit and treatment-related risk necessitates careful patient selection, integration of multimodal biomarkers, and coordinated multidisciplinary workflows [5, 7]. Updated appropriate use criteria (AUC) emphasise that biomarker testing should be embedded within specialist-led diagnostic pathways and applied when results are expected to influence clinical management meaningfully [10].

This transition places new responsibilities on healthcare systems, including the need for earlier diagnosis, biologically based patient stratification, standardised safety monitoring, and the reorganisation of services to support infusion therapies and follow-up. In several countries, these challenges are amplified by marked regional heterogeneity. While some clinical centres for dementia and cognitive decline have already integrated fluid biomarkers, Aβ-PET, advanced MRI, and genetic profiling into routine practice, others face structural and organisational constraints that may limit equitable access. In this setting, the implementation of AATs represents not only a therapeutic innovation but also a critical test of health system readiness to deliver coordinated, biologically driven, and sustainable AD care [5].

## Diagnostic pathways in Alzheimer’s disease

The shift from a symptomatic to a biological definition of AD has fundamentally changed the diagnostic pathways in patients with cognitive decline. AD is now considered a biological continuum in which pathological changes precede clinical symptoms by many years, with diagnosis aimed at identifying pathology at the prodromal and even preclinical stages [11].

Current diagnostic pathways rely on a stepwise, multimodal approach integrating clinical assessment, neuropsychological testing, biomarkers, and neuroimaging. Clinical evaluation remains the entry point, providing the context necessary for biomarker interpretation. Biomarkers are intended to complement clinical judgement by increasing diagnostic confidence and specificity, and their selection should be guided by the clinical syndrome rather than test availability [12]. However, these pathways were not designed with the requirements of emerging AATs in mind and therefore do not yet incorporate the diagnostic precision and biomarker standardisation needed to support treatment eligibility and monitoring.

Fluid biomarkers play a central role in first-line care. CSF and BBB (the latter currently available only in research settings) enable the identification of AD pathophysiology with increasing accuracy and scalability, supporting case finding and triage. However, in a few cases, discordant or inconclusive results may occur, particularly in mixed or atypical presentations, necessitating complementary imaging [12].

Structural MRI remains mandatory throughout the pathway. Beyond excluding alternative causes of cognitive impairment, MRI helps with disease staging and assessment of vascular comorbidity burden with particular emphasis on findings related to cerebral amyloid angiopathy (CAA), a condition with the highest risk of ARIA during anti-amyloid treatment. In the era of AATs, MRI will also serve as a governance role by supporting baseline safety assessment [13].

Aβ-PET is a complementary tool with high diagnostic specificity, especially when first-line assessment with CSF fluid biomarkers is not feasible or yields uncertain, discordant, or biologically ambiguous results. It has been shown that early deployment of Aβ-PET (within 1 month) increases etiological diagnostic confidence by 3.5-fold at 3 months compared with delayed or no Aβ-PET [14]. This effect is consistent across MCI and mild dementia and, to a lesser extent, for subjective cognitive decline, and leads to a faster resolution of undetermined or misclassified diagnoses (from 40 to 11%; *P* < 0.001) [14–16].

Furthermore, owing to their high lipophilicity and the use of modified dual-point acquisition protocols, early-phase Aβ-PET frames with all EMA- and FDA-approved Aβ-PET tracers have been shown to provide information on cerebral blood flow (CBF). Pioneering pathophysiological studies have established that CBF is closely coupled with brain metabolism [17]. Indeed, early-phase Aβ-PET frames provide a cerebral perfusion-weighted signal that correlates with FDG metabolism tomography at the individual level (*r* > 0.72, *P* < 0.001), offering an FDG-comparable map of neuronal dysfunction when Aβ-PET is acquired in dual-phase protocols, while late frames confirm fibrillar amyloid distribution [15, 18]. In this regard, early perfusion imaging remains a promising research tool, as high-level evidence of non-inferiority to FDG-PET (especially in MCI patients) is still lacking due to a shortage of dedicated clinical trials. Accordingly, its routine clinical use and integration should also be weighed against other alternatives. In particular, MRI-based techniques, such as Arterial Spin Labelling (ASL), can provide similar hemodynamic information and might represent an additional option for diagnostic workflows, especially when interobserver agreement and methodological standardisation are further refined [19].

To summarise, amyloid PET is a well-established clinical tool for the biological diagnosis of AD. Given the current need to allocate scanning slots to emerging indications for AAT treatment, these slots should be dedicated specifically to patients with inconclusive CSF results or those who cannot undergo CSF assessment. In this regard, while visual reading may serve as a standalone metric for diagnostic purposes, semiquantification plays an important role in the initial assessment and is a crucial complement as treatment eligibility and monitoring are approached (see below). Furthermore, it is important to distinguish between the diagnostic pathway—aimed at confirming the underlying AD pathology—and the patient selection process for AATs. While Aβ-PET remains the gold standard for both, therapy selection requires additional considerations, including quantification of amyloid burden and assessment of co-pathologies, to optimise the benefit-to-risk ratio.

## Selection of patients eligible for anti-amyloid therapy

Real-world studies indicate that eligibility for AATs varies substantially across clinical settings, diagnostic pathways, and operational criteria. Early reports from specialist centres suggested that a relatively small proportion of patients evaluated met strict trial-based eligibility criteria, with estimates in the single-digit range in some European cohorts and population-based samples [20]. However, more recent analyses from tertiary memory clinics that incorporate structured biomarker workflows indicate that eligibility may be higher when systematic assessment is employed. For example, application of an AT(N)-based framework in an Italian hospital cohort found that approximately 17% of biomarker-positive patients met treatment criteria [21], while Swedish memory clinic data showed that up to 13% of consecutively evaluated patients, and a substantially larger proportion within the MCI/dementia subgroup, could be considered eligible depending on operational definitions [22]. Similar variability has been observed in clinic-based biomarker triage studies, underscoring that eligibility reflects not only disease prevalence but also the structure and implementation of diagnostic pathways [23, 24]. Collectively, these findings suggest that eligibility should be interpreted as a dynamic range likely influenced by clinical infrastructure rather than a fixed limitation.

Patient selection is therefore a multidimensional process integrating disease stage, biomarker confirmation, and individual risk–benefit considerations. While early symptomatic AD presentation defines the target population, clinical criteria alone lack specificity because they overlap with those of other neurodegenerative and vascular conditions. Revised guidelines emphasize that treatment decisions must be based on biological evidence of AD [11].

As previously mentioned, AD pathophysiology can be confirmed using CSF or BBB, which serve as scalable triage tools within specialist pathways [11, 25]. In this setting, fluid biomarkers provide indirect measures of pathology and may yield borderline or discordant results in complex cases [26].

Accordingly, when moving from diagnosis to eligibility, Aβ-PET serves as a high-specificity confirmatory modality, further reducing diagnostic uncertainty and establishing a quantitative baseline that informs treatment planning from multiple perspectives. In fact, PET-derived amyloid burden and distribution support staging decisions, help contextualise safety considerations, and provide a reference against which therapeutic effects can be interpreted. In this regard, a specific metric, the Centiloid scale, has emerged from clinical trials and subsequent recommendations as a standardised and robust way to increase the clinical value of Aβ-PET. Before validation of the Centiloid scale, interpretation of PET scans using different tracers relied on varying cut-offs to define a positive or negative scan, making it challenging to compare across clinics or research studies. Furthermore, the Centiloid scale is not just a binary categorisation of the scans and may support a better stratification of patients who are candidates for treatment. It should be noted, however, that a range in the Centiloids scale—typically between 10 and 25 (or 30) Centiloids—is considered a Grey Zone. When discussing eligibility for treatment, interpreting Centiloid values in the 20–30 range requires significant caution [27]. In this 'grey zone,' measurement noise and biological variability can overlap, meaning that such thresholds should be integrated with visual assessment and clinical context and cannot be used as stand-alone criteria for treatment decisions. On the other hand, existing evidence suggests that an amyloid load exceeding the 60–70 Centiloid threshold may predict significant tau deposition, as assessed by [18F]flortaucipir PET [28, 29]. This finding is relevant because, in trials of donanemab, the magnitude of clinical benefit in the high-tau group was lower than that observed in the low/medium-tau population [3]. Determining or estimating tau deposition is valuable for stratifying patients with different potential benefits from AATs, since better outcomes are predicted in earlier disease stages [3]. Furthermore, in the TRAILBLAZER‐ALZ trial, patient recruitment was based on a specific Centiloids threshold (37 CL), and data from this trial showed that baseline amyloid-PET levels are inversely associated with treatment-related amyloid clearance (TRAC). Specifically, patients achieving full TRAC within 24 weeks had lower initial amyloid levels (92.8 ± 28.7 CL) than those who did not (117.4 ± 33.9 CL) [3].

While the dual assessment of amyloid and tau pathologies offers a robust framework for disease staging, the financial and logistical constraints of multi-tracer imaging limit its scalability in routine clinical practice. Furthermore, although fluid biomarkers—such as p-tau217—effectively reflect secreted hyperphosphorylated tau, they may not yet reliably substitute for the staging of fibrillar tau, the primary driver of neurodegeneration [30]. Consequently, the semi-quantitative analysis of baseline Aβ-PET scans emerges as a critical tool not only for refining patient selection (rule-in/rule-out) but also for optimising prognostic profiling to assess potential benefit prior to therapeutic intervention.

Early-frame Aβ-PET may also provide additional biological context in therapy candidate evaluation, particularly when differential-diagnostic uncertainty persists, or to improve the assessment of the degree of neurodegeneration. This is of particular significance, as tau PET tracers have yet to achieve widespread clinical availability in numerous countries, including Italy [14, 18, 31–33]. The combination of early- and late-phase PET in a single imaging session has been shown to provide complementary information on neuronal dysfunction and amyloid burden, streamlining pre-treatment evaluation without compromising clinical specificity [15, 18].

Overall, eligibility determination requires an integrated, stepwise model in which biomarkers and imaging refine certainty, staging, and safety. Consideration of ARIA risk should be an integral component of this process, supporting responsible patient selection and sustainable therapy implementation. Genotyping (e.g., APOE status) is mandatory for ARIA risk profiling [19, 20]. Clinical trials have shown that APOE ε4 homozygotes (ε4/ε4) experience a higher incidence of ARIA-E and ARIA-H, and are therefore commonly excluded (at least in European Guidelines based on risk–benefit analysis) from treatment initiation under standard monitoring capacity [9, 25, 34]. Recent recommendations for anti-amyloid agents reinforce APOE ε4 homozygosity as a factor for non-eligibility or for reconsidering initiation, particularly in systems where MRI surveillance intervals or neuroradiological capacity are constrained [24].

Structural neuroimaging adds specificity and safety information. Brain MRI is essential for staging and for identifying features associated with increased ARIA risk, including microhaemorrhages and superficial siderosis, particularly when combined with genetic risk factors such as APOE ε4 status. Emerging evidence suggests that amyloid burden, as measured with Centiloids (when higher than 100–110 Centiloids) and also regional occipital Aβ positivity, may carry additional information on ARIA risk [35], supporting individualised safety planning even though MRI remains the reference standard for ARIA detection [9, 24, 25, 33].

In summary, a baseline Aβ-PET assessment based on visual reading and quantification—while not mandatory for treatment eligibility—provides meaningful information that allows for a more comprehensive biological characterisation of patients. This supports a balanced risk–benefit assessment, which is crucial for the introduction of AAT into clinical practice.

## The role of amyloid PET in treatment monitoring

Once therapy is initiated, follow-up Aβ-PET allows objective assessment of pharmacodynamic target engagement and amyloid clearance. This monitoring role is central to treatment strategies such as donanemab, where therapy duration may be guided by biomarker-defined endpoints, but it may also provide biologically relevant information during continuous-treatment approaches such as lecanemab, particularly when clinical evolution or treatment response requires clarification. Within the treatment-related amyloid clearance framework, longitudinal Aβ-PET quantification captures trajectories toward partial reduction or conversion to amyloid negativity, typically observed over 12–18 months, anchoring therapeutic decisions to measurable biological change [3, 4, 31, 36].

AATs consistently induce substantial reductions in cerebral amyloid burden as measured by Aβ-PET, with many treated patients converting from amyloid-positive to amyloid-negative status within 6–10 months [3, 4], supporting Aβ-PET as the only reference modality for treatment monitoring, ideally when baseline amyloid positivity has already been established by fluid biomarkers or prior Aβ-PET imaging. Within the TRAC framework, Aβ-PET-based assessment, particularly when combined with a pre-treatment (baseline) scan, enables high-certainty classification of partial and complete clearance trajectories, with quantification recommended to define the degree of amyloid reduction, target engagement, and treatment response, rather than relying on binary visual interpretation alone [31]. Centiloid values have been extensively used in clinical trials for not only for patient selection but also for treatment monitoring with evidence suggesting that reductions below a threshold of 24–30 Centiloids are associated with clinical benefit [37]. In particular, in the donanemab trials, the criteria reported in the paper to identify patients who had PET evidence of fibrillar amyloid clearance (< 24.1CL) was different from the criteria used to interrupt treatment (one scan < 11 CL or two consecutive scans < 25 CL) [3].

According to the proposed framework, TRAC should be defined using amyloid PET in patients with pre-treatment biomarker confirmation of cerebral Aβ deposition who were subsequently treated with an AAT, emphasising that semi-quantitative Aβ-PET metrics are essential for reliable baseline-to-follow-up comparison and for determining therapeutic monitoring endpoints [31].

Aβ-PET has been proposed to standardise the interpretation of treatment-induced changes, emphasising that post-treatment biomarker status reflects pharmacodynamic effects rather than disease cure [31]. In clinical practice, follow-up Aβ-PET may inform decisions on treatment duration and discontinuation, as operationalised in donanemab trials and under discussion for the long-term management of lecanemab [36].

Compared with fluid biomarkers, which show variable post-treatment changes, amyloid PET remains the most robust tool for assessing target engagement at the individual level [38]. Tau PET may provide complementary insight into neurodegeneration-related changes; however, it remains investigational for evaluating post-treatment alterations in tau pathology and for monitoring significant modifications in the underlying biologically driven disease trajectory [39]. In summary, TRAC can currently be defined only via Aβ-PET. At present, treatment discontinuation has been implemented only for patients treated with donanemab, based strictly on the predefined thresholds used in clinical trials. Although Aβ-PET imaging was performed every six months in donanemab trials—with the majority of patients achieving full TRAC after 12 months—the optimal window for Aβ-PET-guided therapy discontinuation in a real-world clinical setting cannot yet be clearly defined [31].

## Organisational challenges and system-level barriers

The implementation of Aβ-PET within routine AD care exposes a set of organisational and system-level challenges that extend beyond technical feasibility. Although the clinical value of Aβ-PET is well-established, its practical and equitable deployment is constrained by heterogeneity in diagnostic practices, infrastructure, workforce expertise, and service coordination, particularly in healthcare systems characterised by regional heterogeneity.

Real-world data consistently document substantial variability in the organisation and capacity of memory clinics. In Italy, national surveys of Centres for Cognitive Disorders and Dementias (CCDDs) show significant heterogeneity in staffing models, operating hours, availability of biomarkers and imaging, and adoption of integrated diagnostic pathways [16, 32, 40]. Only a minority of CCDDs currently meet the structural, diagnostic, and organisational requirements considered necessary for the safe prescription and monitoring of AATs, with a clear geographical concentration in Northern Italian regions [40]. Expert consensus has emphasised that the introduction of AATs necessitates redefining dementia care models, including stratifying centres by complexity and formally assigning diagnostic and therapeutic responsibilities [33]. In the absence of a uniformly adopted operational framework, this variability risks delaying diagnosis, limiting access, and undermining appropriate patient selection.

From an imaging perspective, lack of standardisation represents a significant barrier. Differences in scanner platforms, tracers, acquisition protocols, and analysis pipelines reduce comparability between centres and over time. Despite the Centiloids scale proving a robust metric in multicentre settings, experience from large multicentre initiatives demonstrates that harmonisation in imaging, from acquisition and quality control to tracer-independent quantification, is essential for reliable longitudinal assessment, but remains difficult to implement outside structured research environments [15].

Capacity constraints further complicate implementation. A national survey in Germany showed that, while expertise and infrastructure for Aβ-PET are broadly available, current clinical volumes remain low and unevenly distributed, with most examinations concentrated in university hospitals [41]. Although overall technical capacity could expand substantially under appropriate reimbursement and organizational conditions, these findings highlight the need for proactive planning to prevent bottlenecks related to scanner availability, trained personnel, and tracer logistics. Similar capacity and distribution challenges are likely to apply across other European healthcare systems.

Additional organizational barriers arise from limited integration between services. Structural MRI, essential for both diagnostic assessment and ARIA risk stratification, is not always readily accessible or temporally aligned with Aβ-PET imaging. Fragmented coordination between neurology, nuclear medicine, radiology, laboratory medicine, and infusion services reinforces siloed workflows rather than the multidisciplinary care models explicitly recommended for the management of patients treated with amyloid-lowering therapies [15, 16, 32, 33, 40].

Workforce limitations represent a further constraint. Interpretation and quality control of Aβ-PET require specialised expertise that is unevenly distributed across regions. Even with automated analysis pipelines, expert oversight remains necessary to ensure data validity and clinical reliability, making scale-up challenging in resource-constrained settings [15, 41]. Filippi and colleagues have further highlighted that ARIA monitoring and management impose additional demands on neuroradiological expertise and MRI capacity, reinforcing the need for designated referral centres and shared protocols [33].

Finally, data governance and interoperability remain insufficient to support longitudinal monitoring and the generation of real-world evidence. The absence of shared registries and integrated data infrastructures limits feedback into clinical practice, health-economic evaluation, and policy planning, and risks perpetuating regional inequities in access to diagnosis and treatment [16, 32, 33, 40].

Overall, national survey data, expert consensus, and multicentre imaging experience converge on the need for coordinated system-level responses. Structured diagnostic pathways, harmonized imaging standards, multilevel network models, and forward-looking capacity planning are essential to ensure that Aβ-PET and AATs can be implemented in an equitable, efficient, and sustainable manner.

## Economic considerations and sustainability

Health systems preparing to deploy AATs must pair clinical effectiveness with economic sustainability [42, 43]. While Aβ-PET implementation is associated with comparatively higher upfront costs than CSF and BBB, its value increases when used as a decision‑enabling step for initiating or discontinuing therapy rather than as a general diagnostic add‑on [44, 45].

Confirmatory testing is economically most valuable when it informs high‑stakes treatment decisions. Economic models show that Aβ-PET, compared with CSF, yields more accurate diagnoses and favourable long-term outcomes at acceptable incremental cost-effectiveness ratios (ICERs) in US contexts [44]. This value arises not only from improved diagnostic accuracy but also from reducing false-positive or uncertain cases that would otherwise lead to inappropriate use of costly therapies, thereby minimizing avoidable monitoring, safety-related imaging, and follow-up associated with mis-initiated treatment. While these benefits are directionally similar in Europe, the magnitude of Aβ-PET’s economic advantage depends on country-specific tariffs, tracer pricing, and pathway design [46].

The emergence of AATs makes this particularly relevant as they require biomarker confirmation to avoid exposing false‑positive or uncertain cases to substantial pharmaceutical and monitoring costs. Aβ‑PET reduces this risk more effectively than CSF or BBB, which have higher rates of inconclusive results [47, 48].

Treatment monitoring also represents an important new dimension. In donanemab clinical programs, Aβ‑PET-confirmed amyloid clearance was used as a criterion for treatment discontinuation, improving the economic efficiency of time‑limited therapy [49, 50].

Finally, observational evidence suggests that a more precise etiological diagnosis supported by Aβ‑PET may influence longer-term outcomes and costs. Aβ‑PET‑enabled diagnosis has been associated with reduced institutionalisation, lower mortality, and improved care planning compared with non‑PET pathways [42, 51].

Overall, the literature suggests that Aβ-PET is least economically favourable when used broadly for diagnostic clarification alone over short time horizons. Still, its value increases when integrated into structured diagnostic–therapeutic pathways that: (i) utilise less‑expensive biomarkers (e.g., BBB or CSF) for triage where appropriate, (ii) reserve Aβ-PET for cases where results influence high‑cost decisions such as treatment initiation or discontinuation, and (iii) support Aβ‑PET‑guided time‑limited therapy, as demonstrated in donanemab trials. When positioned this way, Aβ‑PET serves as both a diagnostic and strategic economic tool in modern AD management [43, 44, 52].

In the setting of AATs, the use of Aβ‑PET as a treatment-monitoring tool—particularly to support treatment discontinuation after documented amyloid clearance, as explored in donanemab trials—may further enhance economic sustainability by limiting unnecessary treatment duration and associated monitoring costs. Although formal cost-effectiveness analyses in this Alzheimer’s setting are not yet available, analogous evidence from oncology indicates that PET-guided identification of non-responders or treatment-related changes can be cost-effective by preventing the continuation of ineffective and high-cost therapies, supporting the broader economic rationale for biomarker-guided treatment decisions [53].

## Final proposals and recommendations

To ensure the effective and sustainable integration of AATs into clinical practice, we propose a strategic restructuring of diagnostic and organisational workflows.

1. *Operationalise a stepwise, multimodal diagnostic pathway*: A shared diagnostic algorithm must be implemented to optimise resource allocation.
  Triage and Confirmation: Utilise scalable BBB and/or CSF as the first-line method for biological confirmation.
  Strategic Role of PET: Reserve Aβ-PET for resolving cases with discordant or borderline fluid biomarker profiles and for patients unable to undergo lumbar puncture.
  Staging and Safety: Shift the focus of Aβ-PET from simple diagnosis to pre-treatment staging, baseline quantification, and profiling of risk/benefit ratio (e.g., stratification of ARIA risk and estimation of amyloid-related neurodegeneration).
2. *Quantitative Standardisation (Centiloids):* A combined visual assessment and Centiloids-based approach is necessary for therapeutic decision-making. Quantitative Aβ-PET metrics should be included in all patients candidate for treatment.
  Adoption of Centiloids: Implement Centiloid scaling to ensure tracer-independent comparability across centres and longitudinal time points.
  Precision: Combine visual reading and semi-quantification to reduce misclassification based on specific thresholds and to objectively document baseline amyloid burden. A thoughtful correlation between the degree of amyloid burden and clinical history can also support decision-making.
3. *Leverage follow-up PET for response-adapted care*: Longitudinal Aβ-PET is the reference standard for assessing pharmacodynamic target engagement.
  TRAC Framework: Use follow-up imaging to define TRAC trajectories (definition of complete clearance based on clinical trial criteria and definition of partial or no TRAC when a baseline scan is also available).
  Sustainability: Align follow-up scans with key management decisions, such as therapy discontinuation, to minimise unnecessary treatment exposure and associated costs and to reduce pressure on the healthcare system and caregivers.
4. *Implement a hub-and-spoke organisational model*: To address regional heterogeneity and capacity constraints, services should be organised into coordinated networks.
  Hub Functions: Centralise high-complexity tasks—including eligibility boards, quantitative Aβ-PET analysis, infusion oversight, and ARIA management—in tertiary centres with advanced capabilities in neuroradiology and nuclear medicine.
  Spoke Functions: Delegate initial triage, screening, and routine monitoring components to local centres to prevent bottlenecks.
5. *Harmonisation and real-world evidence generation*: Systemic barriers regarding data quality and interoperability must be addressed.
  Technical Harmonisation: Standardise acquisition protocols and reconstruction pipelines to ensure data validity across scanner generations.
  Registry Implementation: Establish national or inter-regional registries to prospectively collect data on biomarker profiles, ARIA events, and long-term outcomes. This infrastructure is essential for auditing equity of access and generating real-world evidence for health-economic modelling.
6. *Investment in training and network culture*: Technical upgrades must be matched by workforce development.
  Curriculum: Prioritise training on appropriate use criteria, quantitative PET interpretation, and multidisciplinary ARIA governance.
  Network Integration: Education should extend to territorial services to ensure timely and appropriate patient referral.

A coordinated, biomarker-driven model of care is no longer aspirational but necessary to ensure that therapeutic innovation in AD is matched by diagnostic precision, safety governance, and system sustainability. Aβ-PET provides unique specificity for treatment-enabling decisions and pharmacodynamic monitoring. Establishing shared standards, scalable triage pathways, and prospective evidence generation will determine whether health systems can translate biological insight into meaningful, reproducible, and sustainable patient outcomes.

## Data Availability

Not applicable.
